# Circulating growth differentiation factor-15 concentration and hypertension risk: a dose-response meta-analysis

**DOI:** 10.3389/fcvm.2025.1500882

**Published:** 2025-04-30

**Authors:** Zhengqing Yu, Jianshu Gao, Zhongwei Zhou, Li Li, Sanqiang Hu

**Affiliations:** ^1^ Department of Clinical Laboratory, Binhai County People’s Hospital, Binhai, Jiangsu, China; ^2^ Department of Cardiology, The Yancheng Clinical College of Xuzhou Medical University, The First People’s Hospital of Yancheng, Yancheng, Jiangsu, China; ^3^ Department of Clinical Laboratory, Affiliated Hospital 6 of Nantong University, Yancheng Third People’s Hospital, Yancheng, Jiangsu, China; ^4^ Department of Blood Transfusion, Lianyungang Maternal and Child Health Hospital, Lianyungang, Jiangsu, China

**Keywords:** growth differentiation factor-15, hypertension, prevalence, dose-response relationship, meta-analysis

## Abstract

**Background:**

Growing evidence suggests that growth differentiation factor-15 (GDF-15) may contribute to adverse clinical outcomes, such as major cardiovascular events and all-cause mortality. However, there is little information about its relationship to hypertension. This meta-analysis aimed to quantitatively evaluate the relationship between circulating GDF-15 and hypertension prevalence.

**Methods:**

Databases searched included PubMed, Web of Science, and Embase, from inception to August 2024. The inclusion criteria were studies reporting hypertension prevalence in at least three GDF-15 categories.

**Result:**

A total of 24 studies from 21 articles with 35,904 participants and 23,925 hypertensive cases were included in this meta-analysis. Compared with individuals with a low level of circulating GDF-15, those with high GDF-15 level had a higher prevalence of hypertension [odds ratios (OR) 1.60, 95% confidence interval (CI) 1.37–1.88, *P* < 0.001). In the dose-response meta-analysis, the prevalence of hypertension increased by 24% with every 1 ng/ml increase in GDF-15 (OR 1.24, 95% CI 1.16–1.33, *P* < 0.001). However, the dose-response curve was nonlinear (*P*-nonlinearity < 0.001), plateauing or even decreasing slightly after GDF-15 concentrations reached approximately 5.5 ng/ml. Significant heterogeneity was detected in the pooled analysis and meta-regression analysis suggested that participants’ age and the prevalence of diabetes significantly accounted for the heterogeneity.

**Conclusions:**

Circulating GDF-15 is positively and non-linearly associated with the prevalence of hypertension, with a plateau or slight decline after reaching a certain GDF-15 dose.

**Systematic Review Registration:** https://inplasy.com/inplasy-2023-3-0082/, identifier (INPLASY202330082).

## Introduction

Hypertension has always been a major public health issue that affects over a billion people worldwide (1). Elevated blood pressure is the leading risk factor for cardiovascular diseases (CVD) and chronic kidney disease (CKD) and their associated mortality (2). Unfortunately, the pathogenesis of hypertension is complex and has not yet been fully elucidated (3). Many risk factors contribute to the development of hypertension, including genetic, pathophysiological, environmental and lifestyle factors (4, 5). Thus, it is necessary to identify suitable biomarkers for predicting the development of hypertension.

Growth differentiation factor-15 (GDF-15) is a member of the transforming growth factor β superfamily that is widely expressed in multiple mammalian tissues, including liver, kidney, prostate, intestinal mucosa (6). Evidence suggests that GDF-15 regulates appetite, energy balance, body weight, lipid metabolism, and immune function, and protects the body from oxidative stress, inflammation, and damage (7, 8). GDF-15 is sensitive to external stimuli with a rapid increase in its circulating levels, and therefore it is well-established as a stress-responsive factor (9). Numerous prospective studies have shown that an elevated level of circulating GDF-15 is a powerful predictor of incident adverse clinical outcomes (e.g., major cardiovascular events and all-cause death) (10–12). However, the ability of GDF-15 to predict the risk of developing hypertension in prospective studies has not been well-studied. In a population-based prospective cohort study, circulating GDF-15 was significantly and positively associated with hypertension at baseline examination, and it was also an independent predictor of hypertensive heart failure during long-term follow-up (13). A recent cross-sectional study showed that circulating GDF-15 levels were significantly higher in patients with grade 2 hypertension than those with grade 1 hypertension and healthy individuals (14). These findings suggest that circulating GDF-15 may be used as a candidate predictor for incident hypertension.

Despite the lack of prospective studies exploring the relationship between circulating GDF-15 and the risk of hypertension, the past dozen years have seen numerous studies providing information on hypertension prevalence across different GDF-15 concentration intervals in different populations. In this study, we performed a dose-response meta-analysis based on all eligible studies to quantitatively evaluate the relationship between circulating GDF-15 and the prevalence of hypertension.

## Materials and methods

The current systematic review followed PRISMA reporting guidelines (15), and the protocol was registered on INPLASY (registration number: INPLASY202330082). Due to the fact this was a systematic review, no ethics approval was needed.

### Search strategy

Three databases, including PubMed, Embase, and Web of Science, were systematically searched through August 30, 2024. We developed a search strategy by combining the following medical subject headings and free text words: (“hypertension” or “high blood pressure” or “blood pressure”) AND (“GDF-15” or “growth differentiation factor-15” or “MIC-1” or “macrophage inhibitory cytokine-1” or “NAG-1” or “NSAID activated gene-1”). Additionally, a manual search of reference lists of included articles was conducted to identify additional eligible studies.

### Study selection

The studies were selected according to the PICOS framework with the following criteria: Population: no restrictions; Exposure: stratification into three or more categories based on GDF-15 levels; Comparators: the highest GDF-15 quantile vs. the remaining quantiles or per 1 ng/ml increment; Outcomes: prevalence of hypertension; Study design: no restrictions. Two researchers independently searched the literature using the standardized screening process. They first screened titles and abstracts against predetermined criteria. Then, in the second phase, they thoroughly evaluated the full texts of potentially relevant studies. Discrepancies between reviewers were arbitrated through consultation with a senior investigator to achieve consensus. Exclusion criteria were as follows: (1) there were no circulating GDF-15 categories or they were only divided into two categories, which made it impossible to perform the dose-response analysis; (2) there were no reports of hypertensive cases in each of the GDF-15 categories, or the number could not be calculated; (3) circulating GDF-15 concentrations cannot be converted to ng/ml, or were not expressed as such; (4) the types of studies were conference abstracts, case reports, reviews, comments, or editorials; (5) the samples were overlapped with those from another study; and (6) non-English language articles. We employed the Cohen's Kappa statistic to assess the consistency between the two reviewers in the selection process. A Kappa value of ≤0.2 signified poor consistency, 0.21–0.40 fair consistency, 0.41–0.60 moderate consistency, 0.61–0.80 good consistency, and 0.81–1.00 very good consistency (16).

### Data extraction and quality evaluation

Two investigators extracted the data and assessed the methodological quality independently, and a third investigator resolved disagreements. We extracted data on the first author, publishing year, location, study design, sample type, GDF-15 assay, and the definition of hypertension. Several participant characteristics were also recorded, including the source population, sample size, age of the participants, the percentage of male participants, body mass index (BMI), smoking status, and diabetes prevalence. Additionally, we extracted the number of hypertensive cases and participants, and GDF-15 concentrations by category. In order to evaluate the methodological quality of selected studies, the Newcastle-Ottawa Scale (NOS) was used, which consists of three domains: selection, comparability, and outcome (17). We classified studies scoring 7–9 points as high-quality, 4–6 points as moderate-quality, and 0–3 points as low-quality.

### Statistical analysis

As a first step, a random-effects meta-analysis was conducted to compare hypertension prevalence among individuals with high and low GDF-15 levels. In this analysis, the high layer is defined as circulating GDF-15 levels in the highest tertiles, quartiles, or quintiles, and low layers are the remaining quantiles. Pooled effect sizes were presented odds ratio (OR) and 95% confidence interval (95% CI).

Based on previously reported methods (18, 19), a dose-response relationship between circulating GDF-15 and hypertension prevalence was investigated. Study-specific ORs and 95% CIs were first estimated for every 1 ng/ml increase in GDF-15 concentration in each study, then these effect sizes were pooled using a random-effects model. For this purpose, the median or mean level of GDF-15, the number of hypertensive patients, and the number of participants in each category are required. When GDF-15 dosages were given as a range, we took the middle point. If open ended lowest or highest GDF-15 categories are reported, the midpoint is estimated by assuming that the width of the category equals that of the adjacent one. In the absence of information about the number of hypertensive cases or participants in each GDF-15 category, we assumed that these individuals were equally assigned into each group. We explored the possibility of the nonlinear relationship between circulating GDF-15 and hypertension prevalence by modeling exposure levels using restricted cubic splines with three knots at fixed percentiles (10%, 50% and 90%) of GDF-15 distribution. A nonlinear *P*-value was calculated by determining whether the second spline coefficient of the model was equal to zero.

*I*^2^ statistic was used to assess heterogeneity, where a value of *I*^2^ greater than 50% indicated statistical heterogeneity. In the case of *I*^2^ > 50%, a random-effects model would be used; otherwise, a fixed-effects model would be adopted. Pre-specified subgroup and meta-regression analyses were carried out to evaluate potential sources of heterogeneity. By omitting 1 study at a time, sensitivity analysis was performed. An Egger's test and funnel plot were used to assess publication bias.

Statistical analyses were performed using Stata software (version 15.0; StataCorp LP, USA). For this meta-analysis, we employed the following Stata commands: “metan” for primary effect size estimation, “metabias” for publication bias assessment, “metaninf” for sensitivity analyses, “metreg” for meta-regression, and “glst” for dose-response analysis. *P* < 0.05 was considered statistically significant.

## Results

### Literature search

**Figure 1:**
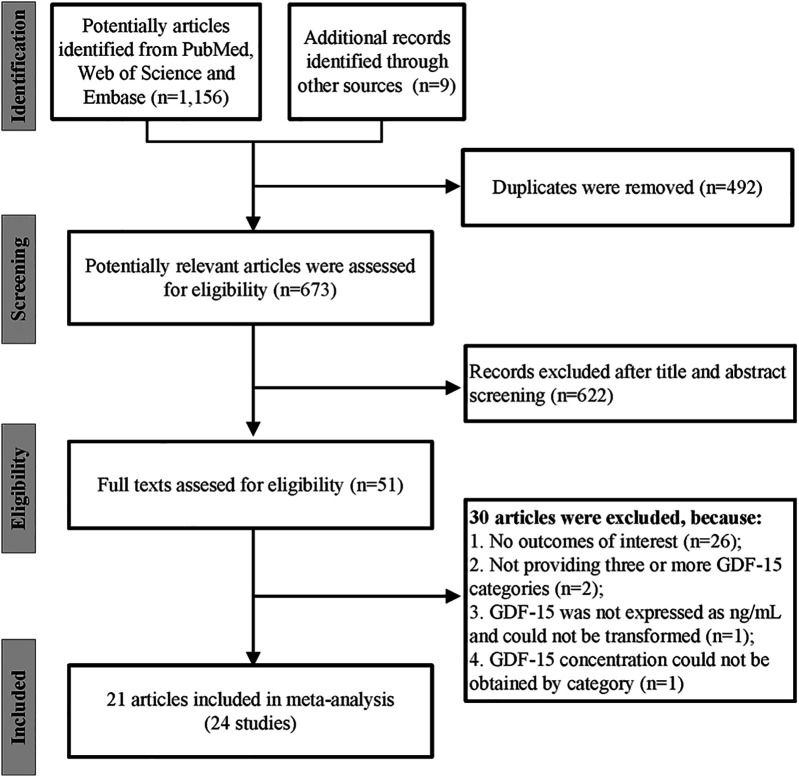
The flow chart of the study selection process.

We initially found 1,156 records in PubMed, Web of Science, and Embase databases, as well as 9 additional records in other sources. After removing 492 duplicates, we screened 673 articles for eligibility. By reviewing titles and abstracts, we excluded 622 records. Of the 51 studies remaining for full-text evaluation, 30 were further excluded: 26 for lack of relevant outcomes, 2 for insufficient GDF-15 categories, 1 for inappropriate GDF-15 units, and 1 for missing categorical data. Finally, a total of 21 articles containing 24 independent studies were included in our meta-analysis (Figure 1) (20–40). The Kappa score for screening titles and abstracts was 0.90, and for full-text screening, it was 0.93, indicating “very good” interrater agreement.

### Characteristics of selected studies

Table 1 summarizes the characteristics of 24 studies from 21 articles. Studies were published from 2009 to 2023, covering 35,904 participants, of whom 21,514 (59.9%) were men. In the 24 studies included, 5 were conducted in Europe (Sweden, Spain, Norway, Germany), 9 in the USA, 9 in Asia (Singapore, Japan, China, Taiwan), and 1 in South Africa. All studies were prospective cohort design, except for one (26) which was a cross-sectional design. The study population involved both clinical and non-clinical populations, including community-dwelling populations, patients with CVD, CKD, and other diseases. The concentration of circulating GDF-15 in the blood was measured by immunoradiometric assay (IRMA) in 2 studies, enzyme-linked immunosorbent assay (ELISA) in 12 studies, electro chemiluminescence immunoassay (ECLIA) in 8 studies, latex turbidimetric immunoassay (LTIA) in 1 study, and Milliplex map kits in 1 study. Half of the studies used serum samples, and the other half used plasma samples. Ten studies gave a definition of hypertension, including systolic blood pressure (SBP) ≥140 mm Hg, diastolic blood pressure (DBP) ≥90 mm Hg, the use of antihypertensives medications, electronic medical records, or self-report. All studies reported the age of participants, while most included studies also reported BMI, smoking prevalence, and diabetes prevalence.

**Table 1:** The general characteristics of the included studies.

Author/year	Region	Study design	Study population	Sample size (% male)	Sample types	GDF-15 assays	Definition of hypertension	Age, year	BMI, kg/m^2^	Smoking, %	Diabetes, %
Lind (2009) (20)	Sweden	Cohort study	Community residents aged 70 years	1,004 (50.0)	Serum	IRMA	SBP ≥140 mm Hg, DBP ≥90 mm Hg, or using antihypertensive drugs	70.0	26.6	10.8	11.8
Bonaca (2011) (21)	USA	Cohort study	Patients after ACS	3,501 (78.9)	Serum	IRMA	N/A	58.1	29.5	36.3	17.4
Rohatgi (2012) (22)	USA	Cohort study	Community-living adults	3,219 (45.0)	Plasma	ELISA	SBP ≥140 mm Hg, DBP ≥90 mm Hg, or using antihypertensive drugs	43.3	29.4	29.9	11.5
Wallentin (2013) (23)	Sweden	Cohort study	Community-living elderly	940 (100)	Plasma	ECLIA	N/A	71.0	26.2	21.0	10.7
Cotter (2015) (24)	USA	Cohort study	Patients admitted for acute heart failure	1,088 (62.6)	Serum	ECLIA	N/A	72.1	29.2	12.8	48.3
Chan (2016) (HFrEF) (25)	Singapore	Cohort study	Patients with heart failure	730 (84.7)	Plasma	ELISA	N/A	59.8	26.0	N/A	55.1
Chan (2016) (HFpEF) (25)	Singapore	Cohort study	Patients with heart failure	186 (43.0)	Plasma	ELISA	N/A	68.3	27.8	N/A	56.5
Martinez (2017) (26)	USA	Cross-sectional study	Patients with COPD	694 (52.9)	Plasma	ELISA	Self-report or using antihypertensive drugs	63.6	27.3	29.8	8.7
Nair (2017) (C-PROBE cohort) (27)	USA	Cohort study	Non-dialysis CKD patients	224 (40.0)	Plasma	ELISA	SBP ≥140 mm Hg, DBP ≥90 mm Hg, or using antihypertensive drugs	58	N/A	12.5	38.8
Nair (2017) (SKS cohort) (27)	USA	Cohort study	Non-dialysis CKD patients	297 (82.9)	Plasma	ELISA	SBP ≥140 mm Hg, DBP ≥90 mm Hg, or using antihypertensive drugs	62	N/A	15.7	56.3
Tuegel (2018) (28)	USA	Cohort study	Non-dialysis CKD patients	618 (60.7)	Plasma	ELISA	N/A	58.5	31.6	13.6	45.1
Sanchis (2019) (29)	Spain	Cohort study	Elderly patients with ACS	208 (54.8)	Plasma	ECLIA	N/A	78.3	N/A	10.1	48.1
Zelniker (2019) (30)	USA	Cohort study	Patients with NSTE-ACS	4,330 (64.8)	Plasma	ECLIA	N/A	64.0	N/A	25.0	32.4
Myhre (2020) (31)	Norway	Cohort study	COVID-19 patients	123 (58.0)	Serum	ECLIA	Electronic medical records	59.6	28.3	4.8	17.1
Oba (2020) (32)	Japan	Cohort study	Patients with cardiometabolic disease	275 (39.0)	Serum	LTIA	N/A	78	23	10.0	52.0
Arnold (2020) (33)	Germany	Cohort study	Patients with osteoarthritis	636 (35.7)	Serum	ECLIA	Self-report	65.0	27.8	11.6	8.8
Wada (2020) (34)	Japan	Cohort study	Patients with suspected or known CAD	2,418 (67.2)	Serum	ELISA	N/A	70.6	24.3	17.7	45.0
Vermeulen (2020) (35)	South Africa	Cohort study	Apparently healthy adults	1,189 (48.2)	Serum	Milliplex MAP kits	N/A	24.6	25.1	23.8	N/A
Negishi (2021) (36)	Japan	Cohort study	Outpatients with cardiovascular risk factors	3,562 (46.0)	Serum	ECLIA	SBP ≥140 mm Hg, DBP ≥90 mm Hg, or using antihypertensive drugs	65.0	24.2	12.1	24.5
Chang (2021) (37)	Taiwan	Cohort study	Hemodialysis patients	170 (54.1)	Plasma	ELISA	N/A	63.6	N/A	N/A	44.7
Echouffo (2021) (38)	USA	Cohort study	Community-living elderly	3,792 (41.0)	Serum	ECLIA	SBP ≥130 mm Hg, DBP ≥80 mm Hg, or using antihypertensive drugs	80.0	28.3	6.8	33.8
Yang (2022) (Diabetic cohort) (39)	China	Cohort study	Patients with ischemic stroke	978 (59.5)	Serum	ELISA	N/A	62.4	N/A	30.7	100.0
Yang (2022) (Non-diabetic cohort) (39)	China	Cohort study	Patients with ischemic stroke	2,023 (66.5)	Serum	ELISA	N/A	62.3	N/A	40.1	0.0
Wang (2023) (40)	China	Cohort study	Patients with CAD	3,699 (72.0)	Plasma	ELISA	SBP ≥140 mm Hg, DBP ≥90 mm Hg, or using antihypertensive drugs	61.3	25.5	45.7	31.9

HfrEF, heart failure with reduced ejection fraction; HFpEF, heart failure with preserved ejection fraction; C-PROBE, clinical phenotyping and resource biobank; SKS, Seattle kidney study; ACS, acute coronary syndrome; NSTE-ACS, non-ST-segment elevation acute coronary syndromes; COPD, chronic obstructive pulmonary disease; CKD, chronic kidney disease; COVID-19, coronavirus disease 2019; CAD, coronary artery disease; IRMA, immunoradiometric assay; ELISA, enzyme-linked immunosorbent assay; ECLIA, electro chemiluminescence immunoassay; LTIA, latex turbidimetric immunoassay; FPG, fasting plasma glucose; BMI, body mass index.

The number of hypertensive cases and participants as well as the concentration of GDF-15 in each category were displayed in Supplementary Table 1, where 13 studies had three GDF-15 categories, and 11 had four categories. The total number of hypertensive cases in all the studies was 23,925.

Based on NOS scale, 2 studies (29, 37) were rated as moderate quality (NOS score = 6), and the remaining 22 were rated as high quality (NOS score ≥7). Details of the appraisal are shown in Supplementary Table 2.

### Hypertension prevalence in high vs. low circulating GDF-15 concentration

**Figure 2:**
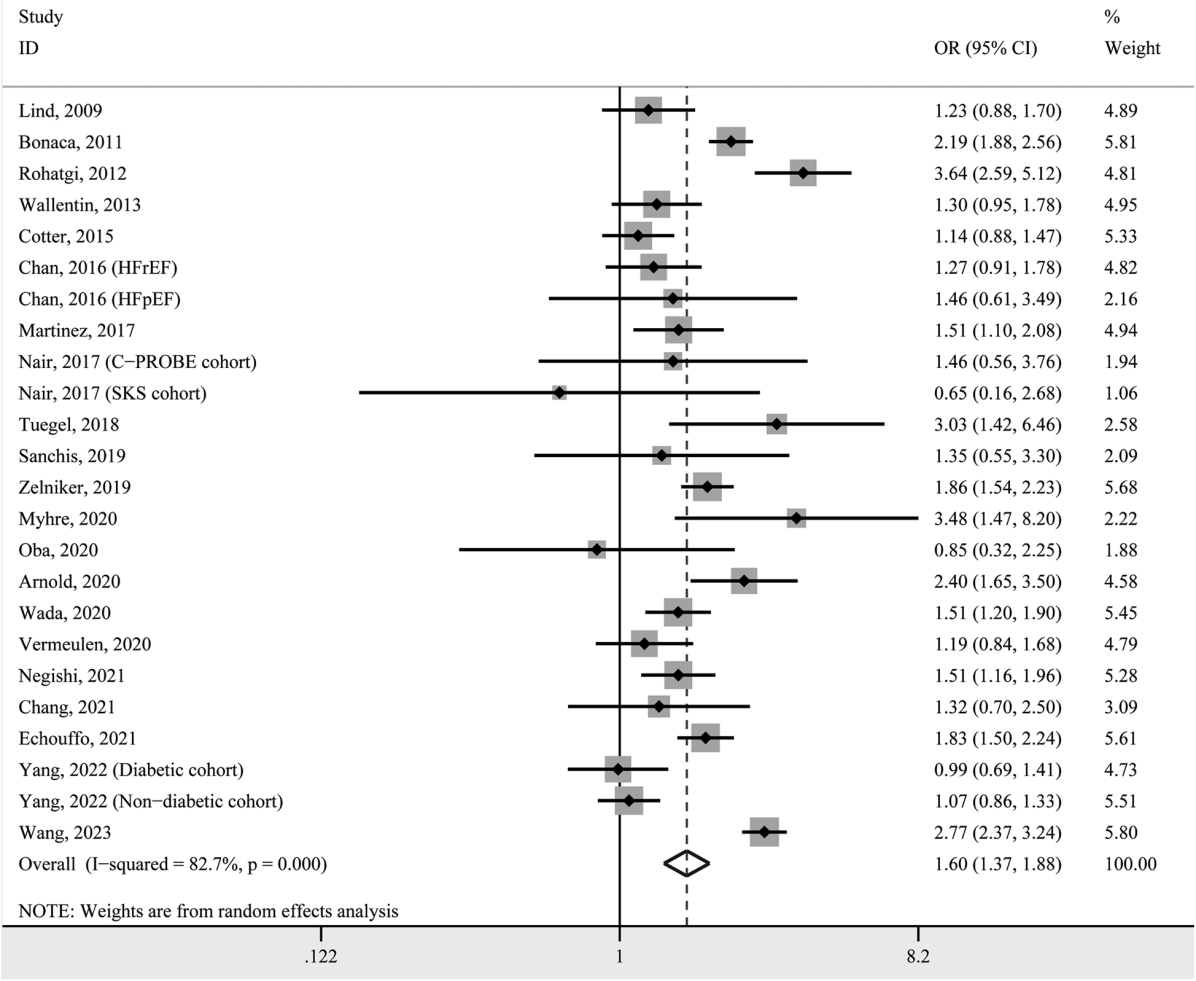
Forest plot of hypertension prevalence in individuals with high GDF-15 concentration vs. those with low GDF-15 concentration. OR, odds ratio; CI, confidence interval.

We first performed a two-class meta-analysis, in which the first highest GDF-15 quantile was classified as the high layer, while the remaining GDF-15 quantiles were categorized as the low layer. Based on the pooled analysis, participants in the high GDF-15 layer were 1.60 times more likely for incident hypertension compared with individuals in low GDF-15 layer (OR 1.54, 95% CI 1.37–1.88, *P* < 0.001; Figure 2). A random-effects model was used due to the significant heterogeneity (*I*^2^ = 82.7%).

### Dose-response correlation between circulating GDF-15 concentration and hypertension prevalence

**Figure 3:**
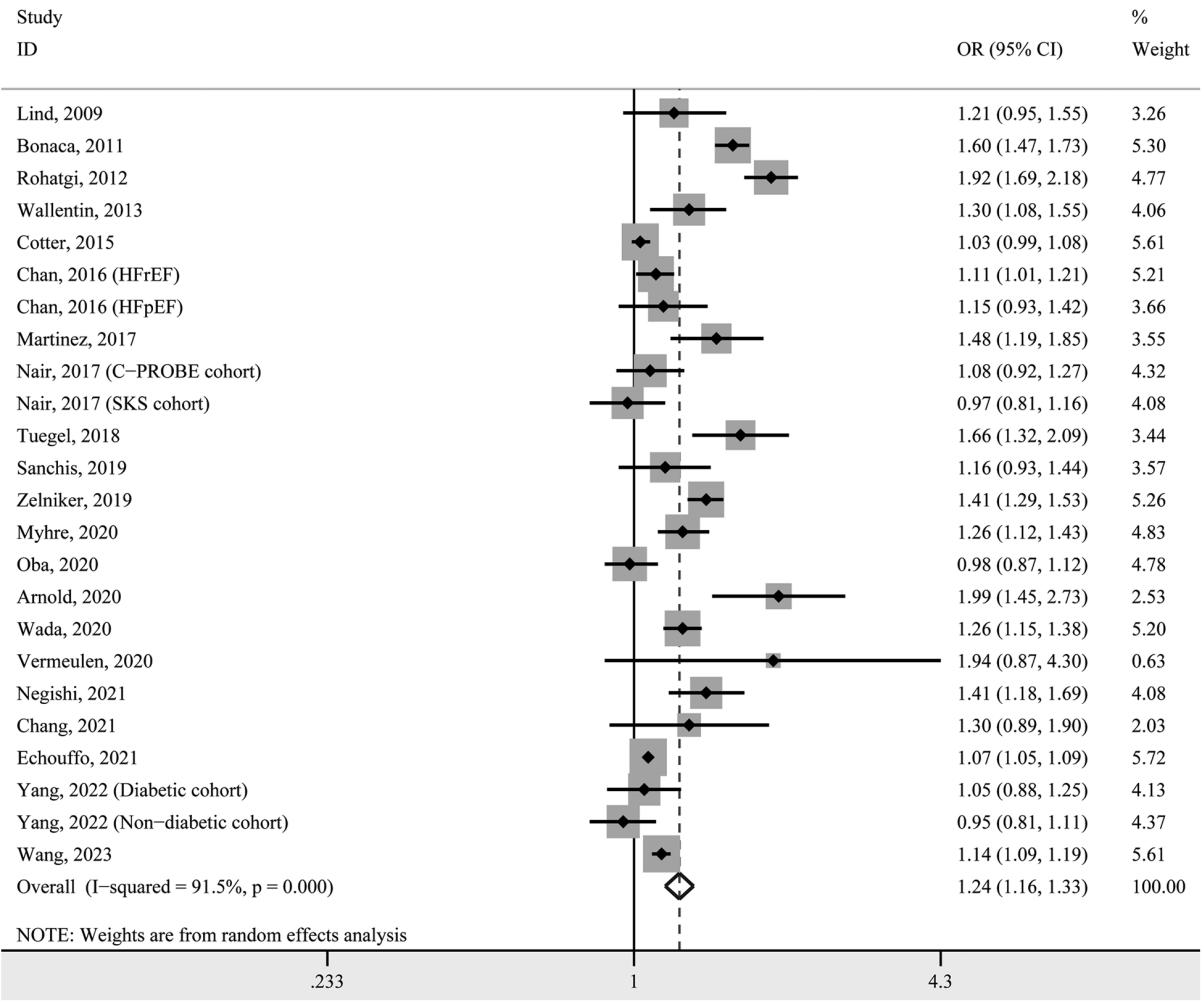
Forest plot for study-specific prevalence of hypertension for per 1 ng/ml increase in GDF-15 concentration. OR, odds ratio; CI, confidence interval.

To obtain a pooled effect size for every 1 ng/ml increase in circulating GDF-15, we calculated study-specific ORs and 95% CIs in each included study and then pooled them. The pooled result indicated that the prevalence of hypertension increased by 24% with each 1 ng/ml increase in circulating GDF-15 (OR 1.24, 95% CI 1.16–1.33, *P* < 0.001; Figure 3). Due to significant heterogeneity (*I*^2^ = 91.5%), these effect sizes were still pooled using the random-effects model.

**Figure 4:**
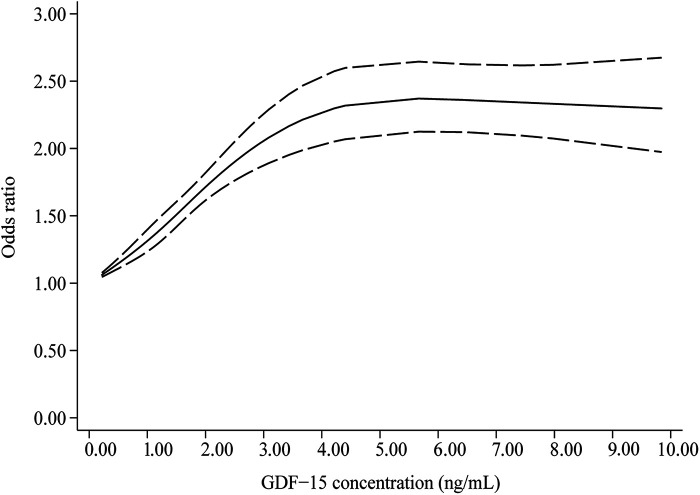
Dose-response correlation between circulating GDF-15 concentration and hypertension prevalence (*P*_non−linearity_ < 0.001).

In Figure 4, the dose-response curve indicates a nonlinear correlation between GDF-15 concentration and the prevalence of hypertension (*P-*nonlinearity < 0.001). As circulating GDF-15 concentration increased, the OR for hypertension prevalence increased, but after reaching approximately 5.5 ng/ml, the dose-response curve plateaued or even decreased slightly.

### Subgroup analysis

Subgroup analysis was performed to assess the sources of heterogeneity, as well as to determine whether a variety of clinical factors affected the dose-response relationship. The following factors were taken into account when stratifying studies, including study areas, source populations, study designs, sample types, and GDF-15 detection methods. In terms of the source population, we divided them into four categories: non-clinical populations (20, 22, 23, 35, 38), patients with severe CVD (21, 24, 25, 29, 30, 34, 39, 40), patients with CKD (27, 28, 37), and patients with other diseases (26, 31–33, 36). Supplementary Table 3 illustrates that heterogeneity persisted across all subgroups, with *I*^2^ statistics ranging from 53.1% to 96.5%. However, the dose-response relationship was not significant for patients with CKD (*P* = 0.120). Nonsignificant dose-response relationships also were observed in 3 subgroups with only 1 study, namely, African participants (*P* = 0.104), GDF-15 detection methods by LTIA (*P* = 0.754), and Milliplex map kits (*P* = 0.104).

### Meta-regression analysis

A meta-regression analysis was carried out with the prevalence of hypertension (OR, each 1 ng/ml GDF-15 increase) as the dependent variable, and several continuity variables including age, the percentage of male sex, BMI, sample sizes, smoking prevalence, and diabetes prevalence as independent variables. Supplementary Table 4 shows that there were negative correlations between the prevalence of hypertension and age (*P* = 0.007) as well as diabetes prevalence (*P* = 0.026). Other independent variables were not significantly correlated with the prevalence of hypertension (all *P* > 0.05).

### Sensitivity analysis and bias of publication

A sensitivity analysis showed that excluding any single study had no effect on the pooled effect size for each 1 ng/ml increase in GDF-15 (Supplementary Figure 1). There was, however, considerable asymmetry in the funnel plot (Supplementary Figure 2) and Egger's test indicated that publication bias may exist (*P* = 0.012).

## Discussion

This study represents the inaugural meta-analysis to quantify the association between circulating GDF-15 levels and the prevalence of hypertension. Our findings initially suggest that elevated GDF-15 levels are associated with a higher prevalence of hypertension. Subsequent dose-response meta-analysis revealed that each 1 ng/ml increment in GDF-15 concentration corresponds to a 24% increase in hypertension prevalence. Nevertheless, the dose-response curve exhibited a plateau or slight decline at higher GDF-15 concentrations, indicating that the prevalence of hypertension does not increase linearly with rising GDF-15 levels. These findings suggest that GDF-15 concentration may elevate as a compensatory mechanism in hypertensive patients. However, this compensatory response appears to have a threshold, beyond which GDF-15 levels cease to increase and may potentially decrease.

Besides being a risk factor for adverse cardiovascular events such as stroke, heart failure and cardiovascular death, hypertension itself is a common cardiovascular disease (41, 42). It is established that obesity, chronic inflammation, oxidative stress and atherosclerosis are relevant to the development of hypertension, beyond its relationship with genetic factors, eating habits, and lifestyle (43, 44). Interestingly, however, although multiple studies have been done to investigate the relationship between circulating GDF-15 and the risk factors associated with hypertension (7–9), little work has been done to explore the relationship between GDF-15 and the risk of hypertension. During the latest years, a number of meta-analyses already summarized the diagnostic and prognostic value of circulating GDF-15 for diverse human diseases. GDF-15, for instance, has been identified as a promising candidate biomarker for gynecological tumors, digestive system tumors, and mitochondrial diseases in several meta-analyses (45–47). It has also been shown that higher concentration of GDF-15 was a significant predictor of adverse cardiovascular events, cardiovascular mortality and all-cause mortality in a variety of acute and chronic diseases (10–12). However, none of the above meta-analyses evaluated the dose-response relationship between GDF-15 and adverse events.

A recent study from our team (48) demonstrated that there is a positive and non-linear association between circulating GDF-15 and CKD progression and poor outcome. Similar to the present study, the linear response was only observed in GDF-15 concentration range of 0–3 ng/ml, but the curve showed a gentle slope over 3 ng/ml. In the prior meta-analysis, we included 7,813 participants, but fewer than half of these were included in the dose-response study. In contrast, we included more studies and samples in our present meta-analysis, which was comprised of 24 studies involving 35,904 participants and 23,925 hypertensive individuals. It is therefore reasonable to believe that the relatively small sample size prevented us from observing a plateau or decline in the prior curve after a specific concentration of GDF-15. The finding also suggests that exogenous GDF-15 supplementation may be advantageous for the prevention or treatment of chronic metabolic diseases, such as hypertension. In fact, this potential therapeutic benefit has been demonstrated in recent murine studies through the administration of GDF-15. In mouse models of non-alcoholic steatohepatitis (NASH), overexpression of GDF-15 was shown to mitigate the progression of NASH, and this mitigation was evidenced by reduced expression of inflammatory and fibrotic genes, as well as decreased levels of liver enzymes, liver weight, and liver triglyceride content (49). Conversely, deletion of the GDF-15 gene resulted in the opposite outcomes (49). The protective effect was also observed in mouse models of diabetic nephropathy, which indicated that renal and systemic inflammation, the AGE/RAGE axis and its downstream inflammatory and adhesion molecules were significantly inhibited when GDF-15 was overexpressed (50). Indeed, GDF-15 has been suggested to be a biologically active protein with therapeutic potential in metabolic disorders, given that it could effectively improve cardiovascular risk factors such as oxidative stress, insulin resistance, and dyslipidemia (51). However, the dose-response curve observed in this study indicates that GDF-15 may have a dual role in hypertension pathophysiology, potentially serving as an adaptive protective mechanism during early disease stages, while chronic exposure to elevated concentrations may induce receptor-mediated pathophysiological alterations. At the molecular level, GDF-15 modulates key biological processes including appetite suppression, energy homeostasis, and vascular remodeling through GFRAL receptor activation (52). Based on established receptor pharmacology principles, prolonged exposure to high ligand concentrations can result in two potential mechanisms: receptor desensitization, which reduces the efficiency of intracellular signal transduction, and receptor occupancy saturation, where further increases in ligand concentration do not elicit additional biological effects (53, 54). The observed plateau phenomenon in circulating GDF-15 levels among hypertensive patients may reflect functional adaptations within the GFRAL receptor system under chronic hyperactivation. These compensatory changes could potentially impair both the endogenous blood pressure-regulating capacity of GDF-15 and the therapeutic efficacy of exogenous interventions targeting this pathway.

Notably, the hypertension cases analyzed in this meta-analysis predominantly represent secondary hypertension arising from comorbid conditions rather than essential hypertension. A substantial proportion of the cohort consisted of CKD patients, where the pathophysiological landscape reveals a complex interplay between GDF-15 kinetics and hypertensive mechanisms. In this population, compromised renal clearance capacity leads to progressive GDF-15 accumulation, which interacts bidirectionally with hypertension drivers such as sodium retention and fluid overload (55, 56). The cytokine demonstrates paradoxical regulatory effects: while its natriuretic properties through renal tubular action theoretically promote blood pressure reduction, concurrent uremic toxin accumulation and persistent volume expansion create counterregulatory hypertensive forces. This pathophysiological interplay may establish a concentration-dependent equilibrium within specific GDF-15 thresholds, clinically manifesting as blood pressure stabilization despite ongoing renal deterioration. Consequently, in CKD-associated hypertension, GDF-15 may exert its influence through multiple pathways, which encompass not only the regulatory mechanisms activated by renal impairment but also potential pharmacological mechanisms involving receptor desensitization and saturation.

In the context of metabolic hypertension, the secretion of GDF-15 exhibits a stress-responsive pattern (9). Obesity, insulin resistance, and inflammatory cascades enhance the transcription of GDF-15 through SMAD-signaling pathways (57), thereby mitigating the elevation of blood pressure induced by metabolic stress. Paradoxically, chronic hypersecretion of GDF-15 can lead to receptor desensitization and may suppress its own synthesis through negative feedback mechanisms. These autoregulatory processes interact with intricate blood pressure regulatory networks, resulting in the nonlinear relationship between GDF-15 levels and hypertension observed in clinical settings. While the dose-response characteristics identified in this study offer new insights into the role of GDF-15 in the pathophysiology of hypertension, the precise mechanisms remain to be elucidated. Future research should focus on the following areas: firstly, the development of GFRAL receptor knockout animal models to test the receptor-dependent hypothesis; secondly, the implementation of prospective cohort studies to elucidate the spatiotemporal relationship between dynamic changes in GDF-15 and blood pressure trajectories; and thirdly, the investigation of the functional heterogeneity of the GDF-15/GFRAL axis in hypertensive patients with diverse clinical phenotypes. These studies are expected to provide a theoretical basis for precise stratified management of hypertension and the development of therapeutic targets.

There is a considerable strength in this meta-analysis because a large number of studies were included, and every study was included in the dose-response analysis, whereas our aforementioned meta-analysis, which included 14 studies, only included a few dose-response studies (48). Another strength is that we explored the sources of heterogeneity using both subgroup analysis and meta-regression, and the latter suggested that age and diabetes prevalence contribute to heterogeneity. Moreover, our sensitivity analysis indicated that the pooled results were robust. There are also several potential limitations to be considered. Firstly, both the clinical and nonclinical populations were included in this meta-analysis, and clinical populations included patients with CKD, severe CVD, and other diseases. In this study, we were unable to determine whether these diseases had an effect on hypertension prevalence. However, in nonclinical populations (20, 22, 23, 35, 38), the prevalence of hypertension increased by 39% with every 1 ng/ml increase in GDF-15, similar to the overall pooled result. Secondly, more than half of the included studies did not give definitions of hypertension. Moreover, some patients self-reported blood pressure data (26, 33), which is likely to be inaccurate and underreported. Therefore, hypertension prevalence might be inflated or deflated in some included studies. Thirdly, according to the asymmetric funnel plot, there may be potential publication biases, which may be explained by the exclusion of non-English articles and grey literature such as conference abstracts. However, it is important to recognize that an asymmetric funnel plot may not solely be attributed to publication bias; significant levels of heterogeneity can also result in such an asymmetry (58). Fourthly, the original studies we selected did not directly investigate the association between GDF-15 and hypertension; instead, they examined GDF-15 in relation to other medical conditions. Thus, the hypertension-related data extracted for this meta-analysis were limited to baseline information—specifically, the number of hypertensive patients across GDF-15 categories. Consequently, we could not adjust for confounding factors in assessing the dose-response relationship between GDF-15 and hypertension. Additionally, circulating GDF-15 levels are influenced by various factors, including physiological (e.g., exercise, diet, and weight changes) and pathological conditions (59, 60). Therefore, the dose-response relationship observed in this meta-analysis may not fully represent the true association. Finally, this study is subject to selection bias, primarily because it included only studies that reported hypertension prevalence with at least three GDF-15 categories. Consequently, the results of the two-class meta-analysis, which compared hypertension prevalence between high and low circulating GDF-15 levels, should be interpreted with caution. Furthermore, selection bias may have been introduced by the inclusion criteria that restricted the analysis to English-language studies and full-text articles.

## Conclusions

This dose-response meta-analysis suggested that circulating GDF-15 is positively and non-linearly associated with the prevalence of hypertension. A slight decreasing trend in the dose-response curve implies that the administration of GDF-15 may be beneficial for preventing or treating hypertension. However, to determine the efficacy and impact of GDF-15 supplementation on hypertension or other chronic diseases in humans, it is essential to conduct prospective studies, including clinical trials.

## Data Availability

The original contributions presented in the study are included in the article/Supplementary Material, further inquiries can be directed to the corresponding author.

## Supplementary material

Supplementary Figure 1 Sensitivity analysis of pooled effect estimates for each 1 ng/mL increase in circulating GDF-15 by excluding single study at a time.

Supplementary Figure 2 Funnel plot for assessing publication bias for each 1 ng/mL increase in circulating GDF-15.
