# Supplementary figures and images for: Circulating growth differentiation factor-15 concentration and hypertension risk: a dose-response meta-analysis
Source: Front Cardiovasc Med. 2025 Apr 30;12:1500882. doi: 10.3389/fcvm.2025.1500882 (PMC12075195; doi:10.3389/fcvm.2025.1500882)

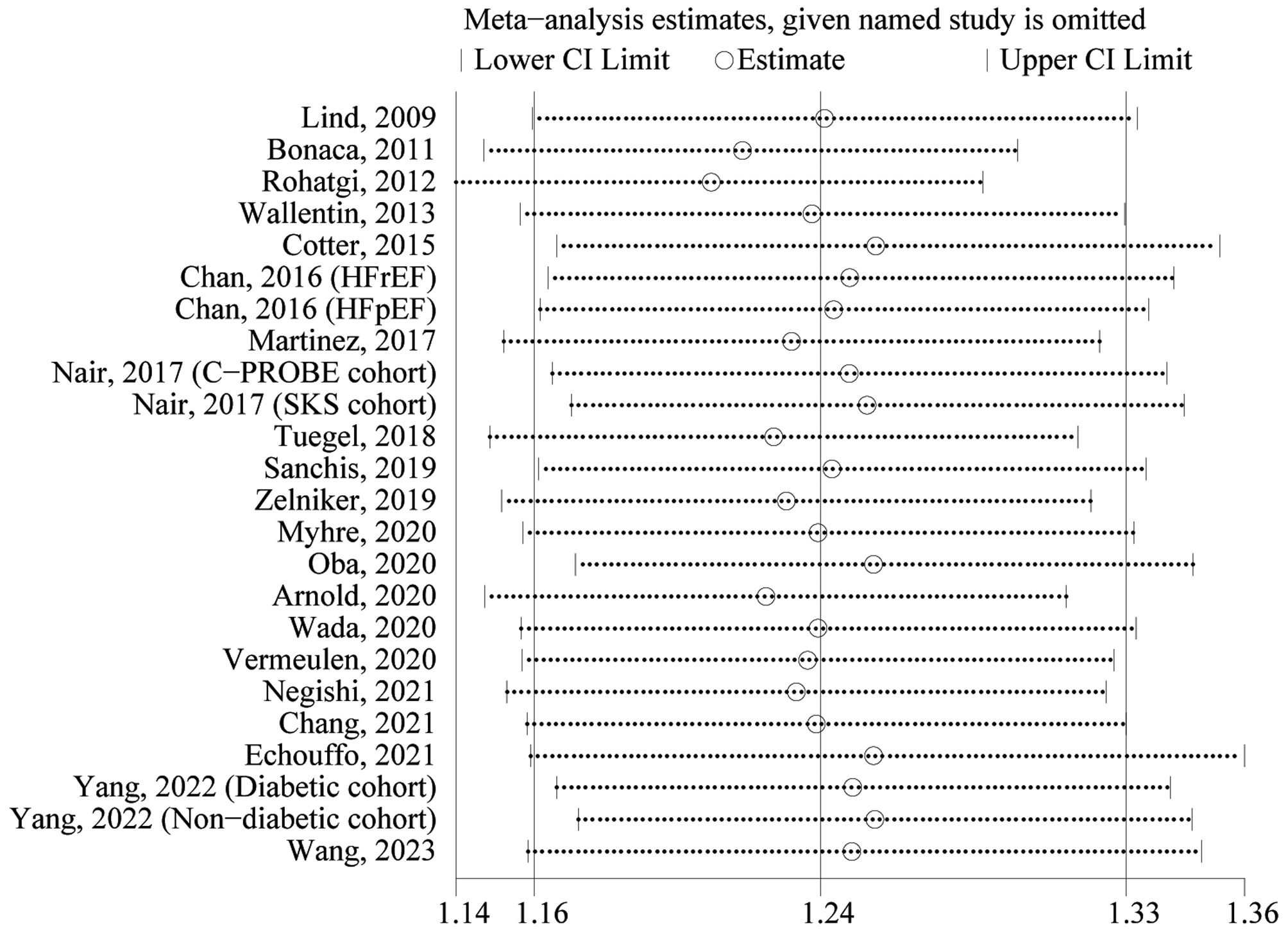

Supplement: Supplementary Figure 1 — Sensitivity analysis of pooled effect estimates for each 1 ng/mL increase in circulating GDF-15 by excluding single study at a time. [file Image1.tif]

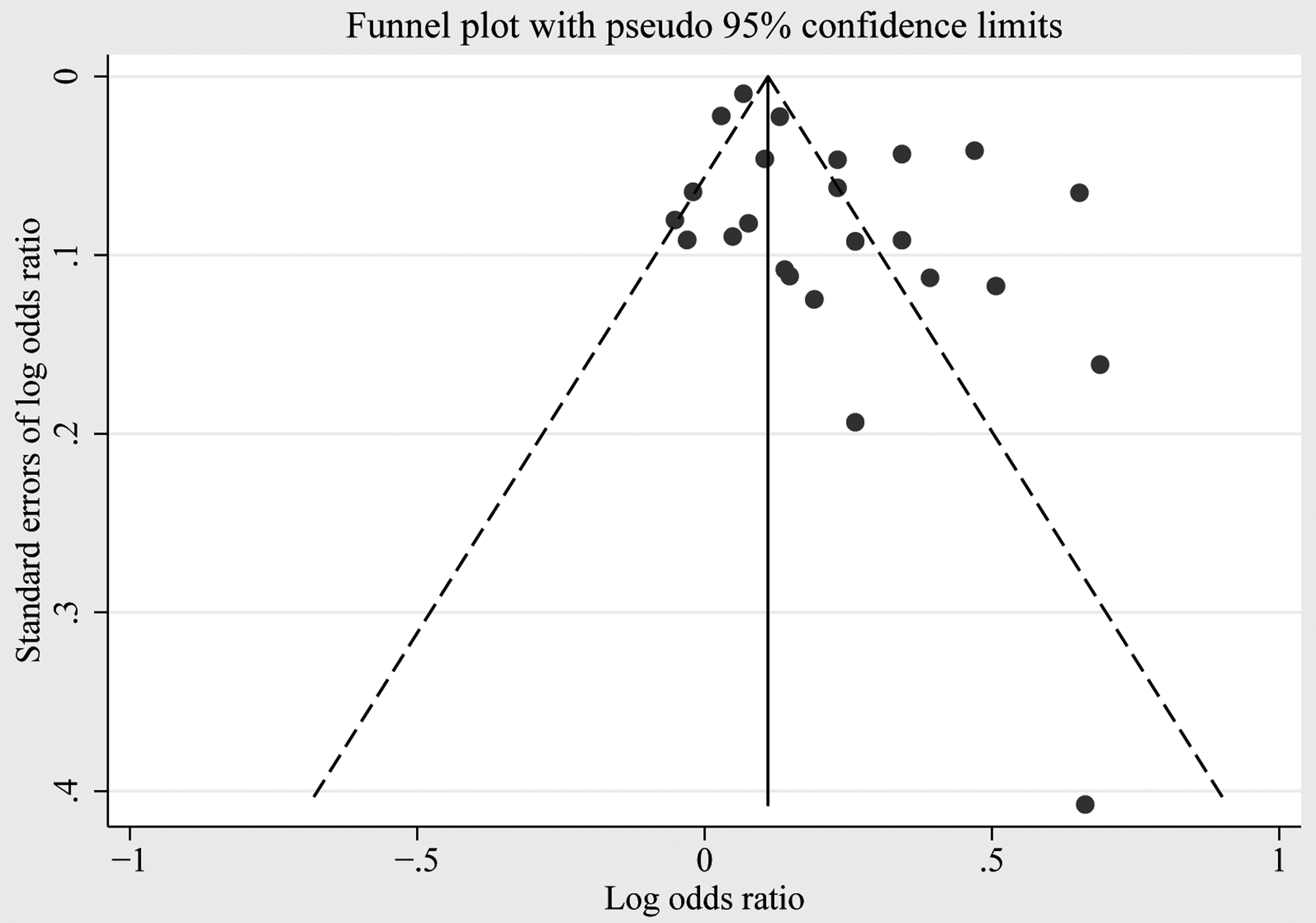

Supplement: Supplementary Figure 2 — Funnel plot for assessing publication bias for each 1 ng/mL increase in circulating GDF-15. [file Image2.tif]
