# Supplementary material for: Circulating growth differentiation factor-15 concentration and hypertension risk: a dose-response meta-analysis
Source: Front Cardiovasc Med. 2025 Apr 30;12:1500882. doi: 10.3389/fcvm.2025.1500882 (PMC12075195; doi:10.3389/fcvm.2025.1500882)
Supplement: Supplementary file 3 [file Table1.docx]

Supplemental Table 1. The number of hypertensive cases and participants, and circulating GDF-15 concentration in each category

| Author/year | No. of  categories | hypertensive cases | Participants | GDF-15 concentration, ng/mL |
| --- | --- | --- | --- | --- |
| Lind, 2009 (20) | 4 | 172/181/184/188 | 250/252/252/250 | <0.948; 0.948-1.134; 1.135-1.390; >1.390 |
| Bonaca, 2011 (21) | 3 | 510/628/593 | 1337/1231/933 | <1.20; 1.20-1.80; >1.80 |
| Rohatgi, 2012 (22) | 3 | 854/156/95 | 2846/295/150 | <1.20; 1.20-1.799; ≥1.80 |
| Wallentin, 2013 (23) | 3 | 213/241/242 | 314/313/313 | <1.307; 1.307-1.720; >1.720 |
| Cotter, 2015 (24) | 3 | 184/196/202 | 362/363/363 | <3.119; 3.119-5.329; >5.329 |
| Chan, 2016 (HFrEF) (25) | 3 | 153/173/176 | 243/243/244 | <1.933; 1.933-3.451; >3.451 |
| Chan, 2016 (HFpEF) (25) | 3 | 45/57/54 | 62/62/62 | <1.933; 1.933-3.451; >3.451 |
| Martinez, 2017 (26) | 3 | 80/107/117 | 231/232/231 | ≤0.966; 0.967-1.438; ≥1.439 |
| Nair, 2017 (C-PROBE cohort) (27) | 4 | 44/50/49/50 | 56/56/56/56 | 0.437-1.307; 1.308-1.938; 1.939-2.891; 2.892-8.444 |
| Nair, 2017 (SKS cohort) (27) | 4 | 69/72/73/70 | 74/73/73/73 | 0.403-1.636; 1.637-2.338; 2.339-3.508; 3.509-9.549 |
| Tuegel, 2018 (28) | 4 | 119/135/144/146 | 154/155/155/154 | <1.280; 1.280-2.019; 2.020-3.063; >3.063 |
| Sanchis, 2019 (29) | 4 | 39/45/45/45 | 52/52/52/52 | <1.750; 1.750-2.432; 2.433-3.746; >3.746 |
| Zelniker, 2019 (30) | 3 | 1580/860/777 | 2286/1104/940 | <1.20; 1.20-1.80; >1.80 |
| Myhre, 2020 (31) | 4 | 3/8/12/16 | 31/31/31/30 | 1.17 (0.99-1.45); 2.27 (1.94-2.51); 3.67 (3.17-4.07); 7.45 (5.25-9.98) |
| Oba, 2020 (32) | 3 | 78/81/79 | 85/86/86 | 0.70-1.44; 1.45-2.13; 2.14-13.82 |
| Arnold, 2020 (33) | 4 | 65/73/82/107 | 158/160/159/159 | <0.78; 0.78-1.005; 1.006-1.279; >1.279 |
| Wada, 2020 (34) | 4 | 421/457/472/493 | 605/604/604/605 | 0.69 (0.55-0.80); 1.10 (1.01-1.21); 1.56 (1.44-1.74); 2.77 (2.25-4.16) |
| Vermeulen, 2020 (35) | 4 | 48/37/58/55 | 338/243/311/297 | 0.219 (0.215-0.222); 0.292 (0.286-0.297); 0.365 (0.359-0.371); 0.521 (0.513-0.530) |
| Negishi, 2021 (36) | 3 | 1051/1083/1107 | 1186/1187/1189 | 0.620 (0.525-0.709); 0.967 (0.873-1.067); 1.582 (1.347-2.044) |
| Chang, 2021 (37) | 3 | 26/32/34 | 56/56/58 | <1.315; 1.315-1.707; >1.707 |
| Echouffo, 2021 (38) | 4 | 665/735/759/806 | 950/947/949/946 | 0.470-1.285; 1.286-1.703; 1.704-2.432; 2.433-17.254 |
| Yang, 2022 (Diabetic cohort) (39) | 3 | 218/263/307 | 271/330/377 | <0.797; 0.797-1.244; >1.244 |
| Yang, 2022 (Non-diabetic cohort) (39) | 3 | 571/523/478 | 728/673/622 | <0.797; 0.797-1.244; >1.244 |
| Wang, 2023 (40) | 4 | 551/584/612/662 | 925/924/925/925 | 0.806 (0.0-1.153); 1.480 (1.151-1.187); 2.332 (1.888-3.043); 4.422 (3.043-5.712) |

HfrEF, heart failure with reduced ejection fraction; HFpEF, heart failure with preserved ejection fraction; C-PROBE, Clinical Phenotyping and Resource Biobank; SKS, Seattle Kidney Study.
