# Supplementary material for: Circulating growth differentiation factor-15 concentration and hypertension risk: a dose-response meta-analysis
Source: Front Cardiovasc Med. 2025 Apr 30;12:1500882. doi: 10.3389/fcvm.2025.1500882 (PMC12075195; doi:10.3389/fcvm.2025.1500882)
Supplement: Supplementary file 4 [file Table2.docx]

Supplemental Table 2. Summary of quality evaluation of the included studies

| **Study** | **Selection** | | | |  | **Comparability** | |  | **Outcome** | | | **Total**  **(out of 9)** |
| --- | --- | --- | --- | --- | --- | --- | --- | --- | --- | --- | --- | --- |
|  | S1 | S2 | S3 | S4 |  | C1 | C2 |  | O1 | O2 | O3 |  |
| Lind, 2009 (20) | 1 | 1 | 1 | 1 |  | 1 | 1 |  | 1 | 1 | 1 | 9 |
| Bonaca, 2011 (21) | 1 | 1 | 1 | 1 |  | 1 | 1 |  | 0 | 1 | 1 | 8 |
| Rohatgi, 2012 (22) | 1 | 1 | 1 | 1 |  | 1 | 1 |  | 1 | 1 | 1 | 9 |
| Wallentin, 2013 (23) | 0 | 1 | 1 | 1 |  | 1 | 1 |  | 1 | 1 | 1 | 8 |
| Cotter, 2015 (24) | 1 | 1 | 1 | 1 |  | 1 | 1 |  | 0 | 1 | 1 | 8 |
| Chan, 2016 (HFrEF) (25) | 0 | 1 | 1 | 1 |  | 1 | 1 |  | 0 | 1 | 1 | 7 |
| Chan, 2016 (HFpEF) (25) | 0 | 1 | 1 | 1 |  | 1 | 1 |  | 0 | 1 | 1 | 7 |
| Martinez, 2017 (26) | 0 | 1 | 1 | 1 |  | 1 | 1 |  | 0 | 1 | 1 | 7 |
| Nair, 2017 (C-PROBE cohort) (27) | 0 | 1 | 1 | 1 |  | 1 | 0 |  | 1 | 1 | 1 | 7 |
| Nair, 2017 (SKS cohort) (27) | 0 | 1 | 1 | 1 |  | 1 | 0 |  | 1 | 1 | 1 | 7 |
| Tuegel, 2018 (28) | 0 | 1 | 1 | 1 |  | 1 | 1 |  | 1 | 1 | 1 | 8 |
| Sanchis, 2019 (29) | 0 | 1 | 1 | 1 |  | 1 | 0 |  | 0 | 1 | 1 | 6 |
| Zelniker, 2019 (30) | 1 | 1 | 1 | 1 |  | 1 | 0 |  | 0 | 1 | 1 | 7 |
| Myhre, 2020 (31) | 0 | 1 | 1 | 1 |  | 1 | 1 |  | 1 | 1 | 1 | 8 |
| Oba, 2020 (32) | 0 | 1 | 1 | 1 |  | 1 | 1 |  | 0 | 1 | 1 | 7 |
| Arnold, 2020 (33) | 1 | 1 | 1 | 1 |  | 1 | 1 |  | 0 | 1 | 1 | 8 |
| Wada, 2020 (34) | 1 | 1 | 1 | 1 |  | 1 | 1 |  | 0 | 1 | 1 | 8 |
| Vermeulen, 2020 (35) | 1 | 1 | 1 | 1 |  | 1 | 0 |  | 0 | 1 | 1 | 7 |
| Negishi, 2021 (36) | 1 | 1 | 1 | 1 |  | 1 | 1 |  | 1 | 1 | 1 | 9 |
| Chang, 2021 (37) | 0 | 1 | 1 | 1 |  | 1 | 0 |  | 0 | 1 | 1 | 6 |
| Echouffo, 2021 (38) | 1 | 1 | 1 | 1 |  | 1 | 1 |  | 1 | 1 | 1 | 9 |
| Yang, 2022 (Diabetic cohort) (39) | 1 | 1 | 1 | 1 |  | 1 | 0 |  | 0 | 1 | 1 | 7 |
| Yang, 2022 (Non-diabetic cohort) (39) | 1 | 1 | 1 | 1 |  | 1 | 0 |  | 0 | 1 | 1 | 7 |
| Wang, 2023 (40) | 1 | 1 | 1 | 1 |  | 1 | 1 |  | 1 | 1 | 1 | 9 |

HfrEF, heart failure with reduced ejection fraction; HFpEF, heart failure with preserved ejection fraction; C-PROBE, Clinical Phenotyping and Resource Biobank; SKS, Seattle Kidney Study.
