# Supplementary material for: Circulating growth differentiation factor-15 concentration and hypertension risk: a dose-response meta-analysis
Source: Front Cardiovasc Med. 2025 Apr 30;12:1500882. doi: 10.3389/fcvm.2025.1500882 (PMC12075195; doi:10.3389/fcvm.2025.1500882)
Supplement: Supplementary file 5 [file Table3.docx]

Supplemental Table 3. Subgroup analyses of dose-response correlation between circulating GDF-15 concentration and the prevalence of hypertension

| Subgroup | No. studies | OR | 95% CI | *P* | Heterogeneity | | |
| --- | --- | --- | --- | --- | --- | --- | --- |
|  |  |  |  |  | *I*^2^ | *P* | |
| Region | | | | | | | |
| Europe | 5 | 1.31 | 1.15, 1.50 | <0.001 | 53.1% | | 0.074 |
| USA | 9 | 1.31 | 1.15, 1.50 | <0.001 | 96.5% | | <0.001 |
| Asia | 9 | 1.13 | 1.05, 1.21 | 0.001 | 64.1% | | 0.004 |
| Africa | 1 | 1.94 | 0.87, 4.31 | 0.104 | / | | / |
| The source population | | | | | | | |
| Non-clinical populations | 5 | 1.39 | 1.02, 1.88 | 0.035 | 95.3% | | <0.001 |
| Patients with severe CVD | 10 | 1.18 | 1.07, 1.30 | 0.001 | 92.5% | | <0.001 |
| Patients with CKD | 4 | 1.21 | 0.95, 1.53 | 0.120 | 78.9% | | 0.003 |
| Patients with other diseases | 5 | 1.35 | 1.10, 1.64 | 0.003 | 84.8% | | <0.001 |
| Study design | | | | | | | |
| Cohort study | 23 | 1.23 | 1.15, 1.32 | <0.001 | 91.7% | | <0.001 |
| Cross-sectional study | 1 | 1.48 | 1.19, 1.85 | <0.001 | / | | / |
| Sample types | | | | | | | |
| Serum | 12 | 1.20 | 1.10, 1.32 | <0.001 | 92.2% | | <0.001 |
| Plasma | 12 | 1.28 | 1.15, 1.43 | <0.001 | 88.1% | | <0.001 |
| GDF-15 assays | | | | | | | |
| IRMA | 2 | 1.43 | 1.09, 1.87 | 0.010 | 77.8% | | 0.034 |
| ELISA | 12 | 1.22 | 1.10, 1.36 | <0.001 | 87.8% | | <0.001 |
| ECLIA | 8 | 1.25 | 1.13, 1.37 | <0.001 | 90.8% | | <0.001 |
| LTIA | 1 | 0.98 | 0.86, 1.11 | 0.754 | / | | / |
| Milliplex map kits | 1 | 1.94 | 0.87, 4.31 | 0.104 | / | | / |

OR, odds ratio; CI, confidence interval; CVD, cardiovascular diseases; CKD, chronic kidney disease; IRMA, immunoradiometric assay; ELISA, enzyme-linked immunosorbent assay; ECLIA, electro chemiluminescence immunoassay; LTIA, latex turbidimetric immunoassay.
