# Supplementary material for: Circulating growth differentiation factor-15 concentration and hypertension risk: a dose-response meta-analysis
Source: Front Cardiovasc Med. 2025 Apr 30;12:1500882. doi: 10.3389/fcvm.2025.1500882 (PMC12075195; doi:10.3389/fcvm.2025.1500882)
Supplement: Supplementary file 6 [file Table4.docx]

Supplemental Table 4. Meta-regression analysis with the prevalence of hypertension (odds ratio, per 1 ng/mL GDF-15 increase) as the dependent variable

| Independent variables | *Exp(B)* | *t* | 95% confidence interval | *P* |
| --- | --- | --- | --- | --- |
| Age | 0.9879 | −2.99 | 0.9799-0.9963 | **0.007** |
| Male percentage | 0.9984 | −0.60 | 0.9929-1.0040 | 0.555 |
| Body mass index | 1.0400 | 1.79 | 0.9925-1.0898 | 0.094 |
| Sample sizes | 1.0000 | 1.29 | 0.9999-1.0001 | 0.210 |
| Smoking prevalence | 1.0002 | 0.63 | 0.9940-1.0108 | 0.632 |
| Diabetes prevalence | 0.9958 | −2.40 | 0.9922-0.9994 | **0.026** |
